# Infection Mechanism of SARS-COV-2 and Its Implication on the Nervous System

**DOI:** 10.3389/fimmu.2020.621735

**Published:** 2021-01-29

**Authors:** Edwin Estefan Reza-Zaldívar, Mercedes Azucena Hernández-Sapiéns, Benito Minjarez, Ulises Gómez-Pinedo, Ana Laura Márquez-Aguirre, Juan Carlos Mateos-Díaz, Jorge Matias-Guiu, Alejandro Arturo Canales-Aguirre

**Affiliations:** ^1^ Unidad de Evaluación Preclínica, Unidad de Biotecnología Médica y Farmaceútica, CONACYT Centro de Investigación y Asistencia en Tecnología y Diseño del Estado de Jalisco (CIATEJ), Guadalajara, Mexico; ^2^ Centro Universitario de Ciencias Biológicas y Agropecuarias (CUCBA), Universidad de Guadalajara, Zapopan, Mexico; ^3^ Laboratory of Neurobiology, Department of Neurology, Institute of Neurosciences, San Carlos Institute for Health Research, Universidad Complutense, Madrid, Spain; ^4^ Biotecnología Industrial, CONACYT Centro de Investigación y Asistencia en Tecnología y Diseño del Estado de Jalisco (CIATEJ), Zapopan, Mexico

**Keywords:** severe acute respiratory syndrome coronavirus 2, coronavirus disease 2019, central nervous system, neurotropism, neuroinvasion, neurological alterations, long-term sequelae

## Abstract

In late December 2019, multiple atypical pneumonia cases resulted in severe acute respiratory syndrome caused by a pathogen identified as a novel coronavirus SARS-CoV-2. The most common coronavirus disease 2019 (COVID-19) symptoms are pneumonia, fever, dry cough, and fatigue. However, some neurological complications following SARS-CoV-2 infection include confusion, cerebrovascular diseases, ataxia, hypogeusia, hyposmia, neuralgia, and seizures. Indeed, a growing literature demonstrates that neurotropism is a common feature of coronaviruses; therefore, the infection mechanisms already described in other coronaviruses may also be applicable for SARS-CoV-2. Understanding the underlying pathogenetic mechanisms in the nervous system infection and the neurological involvement is essential to assess possible long-term neurological alteration of COVID-19. Here, we provide an overview of associated literature regarding possible routes of COVID-19 neuroinvasion, such as the trans-synapse-connected route in the olfactory pathway and peripheral nerve terminals and its neurological implications in the central nervous system.

## Introduction

The severe acute respiratory syndrome coronavirus 2 (SARS-CoV-2) is a positive single-stranded RNA coronavirus responsible for the severe pneumonia coronavirus disease 2019 (COVID-19), as the World Health Organization named in February 2020. As of December 2020, the World Health Organization reported more than 71 million confirmed cases and more than 1.6 million deaths worldwide. SARS-CoV-2 is the seventh member of the coronavirus family that infect humans. Among them, NL63-CoV, HKU1-CoV, 229E-CoV, and OC43-CoV, typically cause common cold symptoms, while SARS-CoV, MERS-CoV, and now the SARS-CoV-2 are responsible for the SARS pandemic in 2002 and 2003, MERS in 2012 and the current COVID-19 pandemic, respectively (1).

SARS-CoV-2 is a betacoronavirus that shares almost 80% sequence identity with SARS-CoV and 50% sequence identity with MERS-CoV (2). Similar to SARS-CoV, SARS-CoV-2 binds to the enzymatic domain of the angiotensin-converting enzyme 2 (ACE-2) receptor exposed on the surface of several cell types, including alveolar cells, intestinal epithelial cells, endothelial cells, kidney cells, monocytes/macrophages, as well as neuroepithelial cells and neurons (3, 4). After spike (S) protein binding to ACE-2 receptor, a subsequent cleavage by transmembrane protease serine 2, cathepsin, or furin, probably induces the endocytosis and translocation of SARS-CoV-2 into endosomes (5, 6), or a direct viral envelope fusion with host cell membrane for cell entry (7). Interestingly, SARS-CoV-2 *in silico* modeling shows a highly structural sequence similarity of 74-79% with SARS-CoV; however, SARS-CoV-2 exhibits some differences associated with a higher binding affinity ACE-2 receptor (8). It has been suggested that the SARS-CoV-2 S protein is slightly more positively charged than SARS-CoV, and the ACE-2 binding interface has a negative electrostatic potential. This electrostatic difference allows a stronger interaction between these two proteins (9, 10). Therefore, this increased binding affinity may promote high virulence of SARS-CoV-2 (11, 12).

COVID-19 symptoms include mild-to-medium fever, cough, diarrhea, fatigue, and dyspnea, progressing to acute respiratory distress syndrome (13). In addition to systemic symptoms, patients also can experience neurological affectation, including headache, dizziness, hypogeusia, hyposmia, myalgia, ataxia, and seizures (14, 15). There are reports of brain edema, partial neurodegeneration, even encephalitis in severe cases of COVID-19 (13, 16, 17). To date, there is no described direct mechanism of SARS-CoV-2 neuroinvasiveness (18). However, it is known that coronaviruses are not always limited to the respiratory system, but they can reach the central nervous system (CNS), inducing neurological impairments (19). This neuroinvasive capacity has been well demonstrated for most beta coronaviruses, including SARS-CoV (20), MERS-CoV (21), 229E-CoV (22), OC43-CoV (23), and HEV (24). Although the SARS-CoV-2 neuroinvasion mechanism remains unknown, considering the high similar viral sequence and infection pathways reported from other betacoronavirus (i.e., SARS-CoV), a similar pathogenic process may be applicable for SARS-CoV-2 (19).

## Neuroinvasiveness Pathways

Despite being a highly protected system with multilayer barriers, the CNS can be reached by some viruses that can infect neurons and glial cells (25). A significant evidence body demonstrates that coronaviruses may reach the CNS inducing neurovirulence (26). Nonetheless, coronaviruses’ determinant route to infect the CNS has not been fully described (27). Usually, the viral infection begins in the peripheral tissues with a subsequent spreading to peripheral nerves and, finally, the CNS (28). This process may explain neurological lesions’ presence, particularly demyelination (29). Moreover, bypasses peripheral barriers, such as the blood-brain barrier (BBB), would be another mechanism (28). In the case of SARS-CoV-2 infection, hyposmia is denoted as one frequent symptom (16), indicating an olfactory dysfunction probably due to neuronal and non-neuronal cells infection in the olfactory system and the involvement of cranial nerves (30, 31). In this way, the CNS invasion, specifically the respiratory center in the medulla and pons, may promote acute respiratory distress in patients with COVID-19 (32).

**Figure 1:**
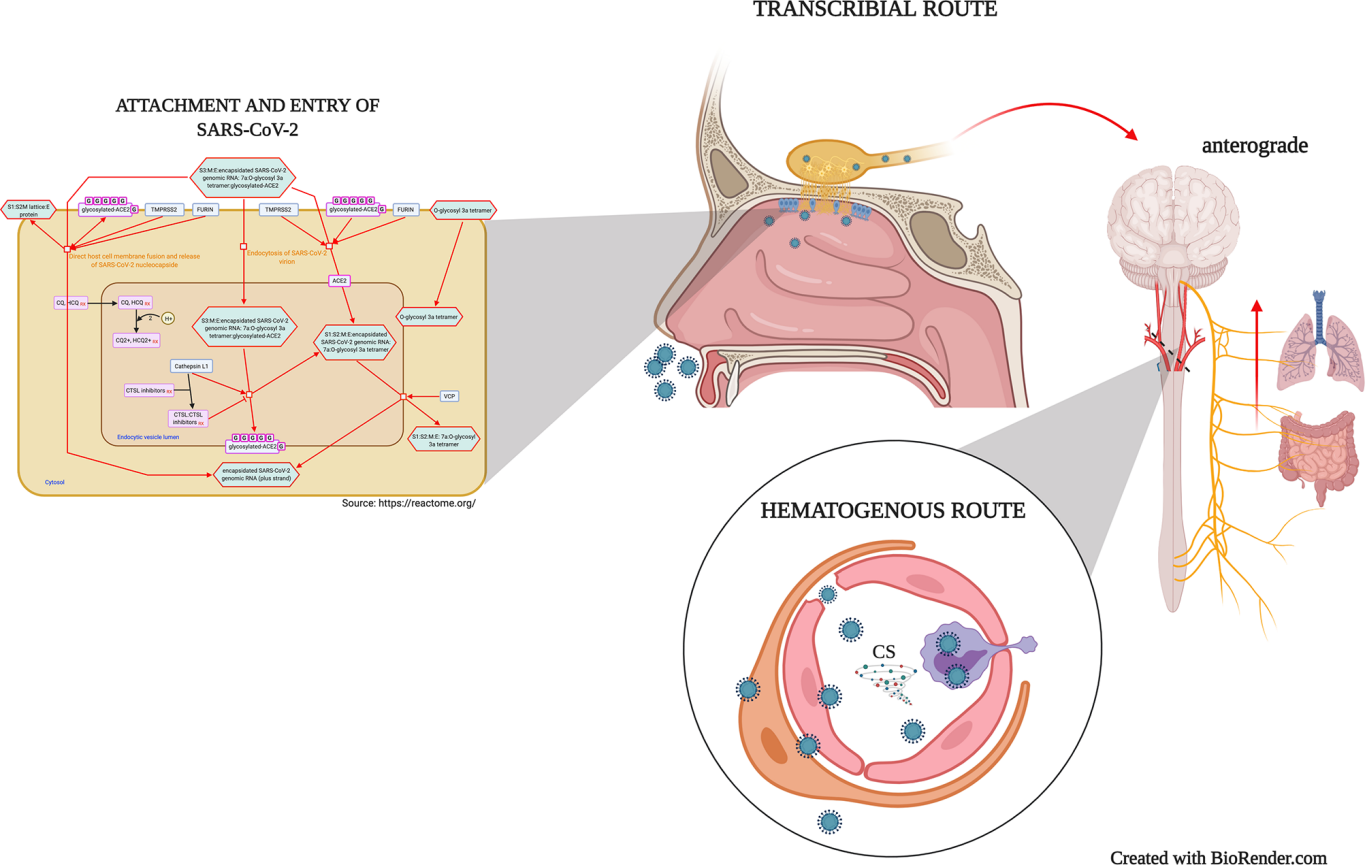
Mechanisms of virion attachment/infection and possibles routes of SARS-CoV-2 neuroinvasiveness. SARS-CoV-2 spike protein interaction with the ACE-2 receptor may promote a direct host cell membrane fusion and the virion nucleocapsid release or induce a Furin/TMPRSS2-mediated endocytosis. Once in the epithelial cells, SARS-CoV-2 spreads to CNS through peripheral neurons in the olfactory epithelium and the second-order neurons along the olfactory nerve (Transcribial route). Similarly, the virus may use different peripheral nerves such as the vagus nerve, which afferences from the lungs and gut reach the brainstem. In the hematogenous route, the viremia induces the infection and viral transcytosis across vascular endothelial cells, as well as leukocytes infection and mobilization towards the BBB, and in several cases, the BBB disruption. CS, Cytokine storm.

Although experimental evidence regarding SARS-CoV-2 neuroinvasiveness is still lacking (33), *post-mortem* studies evidenced the virus’s presence in the brain microvasculature, cerebrospinal fluid, even neurons (4, 26, 34). Also, studies demonstrated that the ACE-2 receptor is expressed on neuron and glial cells of structures such as the olfactory epithelium, cortex, striatum, substantia nigra, and the brain stem (35), supporting the SARS-CoV-2 potential to infect cells throughout the CNS. Therefore, there are suggested mechanisms for coronaviruses neuroinvasion (**Figure 1**), including the neuronal anterograde and retrograde spreading in the transcribial route (8, 16) and (19, 33) the hematogenous route (36). The neuronal retrograde/anterograde transport and the trans-synaptic transfer are supported by *in vitro* studies where the SARS-CoV-2 is detected within neuronal soma and neurites using human brain organoids (31, 37).

## Transcribial Route and Neuronal Transport Dissemination

Growing evidence shows that some coronaviruses first invade peripheral nerve terminals, then are anterograde/retrograde spread throughout the CNS *via* synapses (19, 24, 38), a well-documented neuroinvasive route for coronaviruses such as HEV67 (24, 39) and OC43-CoV (23). Among the peripheral nerves, the olfactory nerve is considered one of the strongest candidates for SARS-CoV-2 dissemination into CNS because of its close localization to olfactory epithelium (27). The olfactory epithelium cells highly express the ACE-2 receptor and the TMPRSS2, necessary for viral binding, replication, and accumulation (40). Recent studies found that neuropilins also have an essential role in cell infectivity. Interestingly, while neuropilin-1 alone promotes SARS-CoV-2 entry and infection, its coexpression with ACE-2 and TMPRSS2 markedly potentiates this effect (41). Similar to ACE-2 and TMPRSS2, the neuropilins are expressed abundantly in the respiratory and olfactory epithelium, becoming the nasal cavity epithelium, a key infection site for CNS infection (8, 41). A recent study presented evidence of SARS-CoV-2 entrance to CNS by crossing the neural-mucosal interface with subsequent penetration of defined neuroanatomical areas receiving olfactory tract projections, including the primary respiratory and cardiovascular control center in the medulla (42). Here, SARS-CoV-2 RNA and characteristic CoV substructures were detectable in the olfactory epithelium and olfactory mucus cells. A subsequent colocalization study within the olfactory mucosa using neuronal markers revealed a perinuclear S protein immunoreactivity in TuJ1^+^, NF200^+^, and OMP^+^ neural cells (42). In this way, SARS-CoV-2 may penetrate the CNS throughout the olfactory receptor cells that pass the cribriform plate contacting second-order neurons of the spherical glomeruli (32, 43). This passage of the olfactory nerve *via* the cribriform plate of the ethmoidal bone was termed transcribial route.

During HEV67 infection, the first coronavirus reported invading porcine brains; the nasal mucosa, lung, and small intestine are first infected. Then is spread to medullary neurons *via* peripheral nerves using the clathrin-mediated endocytic/exocytic system (24, 44). In OC43-CoV infection, a human coronavirus sharing more than 91% homology with HEV67 (45), once in the olfactory bulb, this coronavirus can disseminate to the cortex and other regions including the hippocampus and spinal cord (23, 46). In MHV-CoV infection, the MHV-CoV also accesses the CNS through the olfactory nerve and disseminates it to the limbic system and brainstem (7, 47). The cortical areas with MHV-CoV persistence are associated with demyelinating lesions due to the trafficking and accumulation of T cells and macrophages that participate in myelin destruction (48).

In the intranasal administration of SARS-CoV and MERS-CoV into transgenic mice, the viral CNS invasion is possible through the transcribial route, gaining direct access to the olfactory bulb, and then spreading to the thalamus and brainstem (21, 49). However, the exact mechanism of early CNS access is still unclear. In this context, it has been suggested that SARS-CoV-2 might spread from the olfactory epithelium to the olfactory bulb towards the olfactory nerve, employing the endocytosis/exocytosis system for transsynaptic transfer (34, 50).

In addition to the transcribial route and the olfactory nerve, the virus may use other peripheral nerves such as the vagus nerve, which lungs and gut afferents reach the brainstem (32, 51). The gut-brain axis is a key component involved in disorders that affect the CNS (52). Interestingly, SARS-CoV-2 was detected in COVID-19 patient feces (53). A recent *in vitro* study demonstrated the SARS-CoV-2 capacity to infect human intestinal epithelium (54). Moreover, it has been reported the anterograde and retrograde viral transmission from duodenal cells to brainstem neurons (55). Therefore, it is possible that upon enterocyte SARS-CoV-2 infection, a further transmission to glial and neuronal cells within the enteric nervous system could reach the CNS *via* the vagus nerve (27, 51). In this line, different sets of data demonstrated that initial lung SARS-CoV infection leads to a secondary viral spreading to the brain, particularly thalamus and brainstem regions such as medullary nuclei of the dorsal vagal complex (56). Similarly, Matsuda et al., 2004 reported the influenza A virus spreading from the respiratory tract to the vagal ganglia through the vagus nerve, suggesting a possible transmission from the respiratory mucosa to the nucleus of the solitary tract and the nucleus ambiguous in the brain stem by vagal dissemination (57). However, evidence regarding the enteric nervous system and the SARS-CoV-2 vagus nerve dissemination is almost null, and further research is required.

## Hematogenous Route

The infection and damage of cells of epithelial barriers allow the virus entrance to the bloodstream and lymphatic system, spreading to multiple organs, including the brain (50). Specifically, the BBB is one of the most frequent viral entry routes to the CNS (58). In this way, there are two possible mechanisms for SARS-CoV-2 spreading, which involves the circulation of viral particles into the bloodstream (25, 33): the infection and viral transcytosis across vascular endothelial cells, and the leukocytes infection and mobilization towards the BBB, a well-described mechanism termed Trojan horse (59).

In the first scenario, the virus in peripheral circulation and the sluggish blood flow within the microvasculature appear to be responsible for the binding enhancement of S viral protein and ACE-2 receptor in the capillary endothelium, promoting the viral transport across the basolateral membrane (8, 60). A structural analysis reported that viral-like particles were actively budding across brain capillary endothelial cells, suggesting the hematogenous route as the most probable pathway for SARS-CoV-2 entry (4). Moreover, an *in vivo* research of MERS-CoV tropism demonstrated a virus’s bloodstream circulation followed the endothelial infection (61). Tseng et al. reported a low-level viremia and brain detection of high viral titers two days after intranasal inoculation of SARS-CoV, supporting the virus transmission through the hematogenous route (62). Additionally, SARS-CoV-2 triggers a systemic inflammatory response due to the cytokine storm, with remarkable BBB permeability effects (58, 63). The disruption of this barrier may result in the viral and infected immune cell entry, promoting a further inflammatory response enhancement (64).

In contrast, peripheral lymphocytes and macrophages’ possible infection allows their use as dissemination vehicles facilitating the pass across BBB, meninges, and choroid plexus (58, 65). Interestingly, the coronavirus ability to infect leukocytes (mainly monocytes/macrophages) has been reported in the 229E-CoV and SARS-CoV (66, 67), but only in 229E-CoV has reported the activation of chemokine secretion (67). This trojan horse mechanism generally involves the extravasation of infected leukocytes into meninges and the cerebrospinal fluid (68). However, compelling evidence for immune cell infection by SARS-CoV-2 is still unclear so far.

## Short- and Long-Term Neurological Manifestation of COVID-19

To date, it has been widely described that a broad spectrum of virus infection can spread through the body and eventually reach and affect the mammalian peripheral nervous system (PNS) and CNS when optimal conditions exist (28). Though coronaviruses are mainly associated with upper and lower respiratory disease, their particular neuroinvasive potential is associated with remarkably neurological affections. The hypoxia promoted by respiratory distress has been associated with disturbed brain metabolism and a subsequent neurological manifestation (36). Notwithstanding, there is still a debate regarding if the neurological manifestations are a primary neurologic symptom or secondary consequences of acute respiratory distress syndrome. The evidence supports the neuroinvasive and neurotropism and possible long-term neurological sequelae of coronaviruses, including SARS-CoV and MERS-CoV (69).

Regardless of neuroinvasiveness mechanisms, emergent data from case reports and clinical studies demonstrated that COVID-19 patients exhibit some CNS and PNS complications, ranging from mild to fatal incomes. The most frequent neurological symptoms are mostly nonspecific in the short-term, such as loss of smell and taste, headache, malaise, myalgias, and dizziness. In contrast, moderate-to-severe cases developed acute cerebrovascular diseases, impaired consciousness, and skeletal muscle injury (70). Indeed, these manifestations can be considered a direct virus effect in the CNS (19, 71).

Unfortunately, recovery of the acute infection does not promise a full viral clearance, and if the infection becomes chronic, it may result in long-term sequelae, including chronic neurological impairment (36). Some studies reported the coronavirus persistence in the CNS and some neurologic and tissular affections (69). In mice surviving acute encephalitis caused by OC43-CoV, the viral RNA could be detected even six months post-infection; in correlation with the viral persistence, these mice also display a reduced locomotor activity, subjacent decreased density of hippocampal layers and gliosis (72). Similarly, the RNA of MHV-CoV is detectable in the brain, even 10–12-month post-infection. Surprisingly, the chronic-CNS demyelination persists as late as 90 days post-infection to scattered demyelinated axons at 16 months after infection (73). Interestingly, case reports support that neurotropic viral infection promotes an exacerbated inflammatory response leading to encephalitis or CNS-target autoimmune (i.e., demyelination) response in COVID-19 patients (29, 58).

Guillain-Barre and Miller-Fisher cases are reported without SARS-CoV-2 detection in cerebrospinal samples, supporting the inflammatory response’s role in neurological manifestations (47, 74). Whether the dysregulated immune response remains after the illness resolution, neurological disorders can be developed, including dementia, depression, and anxiety (56, 75). Furthermore, the hypoxia and cerebrovascular diseases reported in COVID-19 patients, particularly encephalitis and stroke, are expected to produce permanent or at least long-term neurological impairments (76).

Although a direct association between SARS-CoV-2 and cognitive impairment is still not correlated, the viral neurotropism and the already reported neurological manifestations support this possible association. A cohort study reported cognitive complaints after SARS-CoV-2 infection, specifically between 10 and 35 days after hospital discharge (71). Here, oxygen therapy and headache were the main variables strongly related to poor performance in neuropsychological tests, indicating cognitive deficits related to attention, memory, and executive function. A case series report evidenced a marked cognitive impairment independent of delirium (4AT score for delirium was unobtrusive) in severely affected patients after 4 to 5 weeks of acute disease onset (47). Another cohort study reported an altered mental status, reflecting neurological and psychiatric, such as encephalopathy, encephalitis, psychosis, and dementia-like syndrome in patients from 23–94 years old; however, cerebrovascular events predominated in older patients (77).

Currently, additional neurological complications reported in other coronaviruses infections may be applicable for SARS-CoV-2. However, the precise and well documentation of neurological symptoms, comprehend the immune response, and the direct impact of brain infection is still needed to better prognosis and prevent long-term effects of SARS-CoV-2 infection.

## Conclusion

Regardless of the different routes of neuroinvasion, it has been demonstrated that SARS-CoV-2 affects the CNS. As overwhelming proof, there is the isolation of SARS-CoV-2 from cerebrospinal fluid, the colocalization within the olfactory system, the ultrastructural evidence of frontal lobe budding SARS-CoV-2, and the list of neurologic manifestations in the COVID-19 patients. Unfortunately, it is unclear if the neurological symptoms of COVID-19 result from cytokine storm-induced neuroinflammation or some brain areas’ infection. Nevertheless, the CNS and immune system involvement might have remarkably neurologic long-term consequences, including the development of neuropsychiatric disorders. Therefore, the awareness of the CNS invasion pathways, the degree of CNS and PNS involvement, and the time course of the viral spreads throughout the nervous system will help comprehend the pathological consequences better and improve the treatment’s diagnostic criteria of possible neurological sequelae.

## Author Contributions

ER-Z, MH-S, BM, UG-P, AM-A, and AC-A: Equal contribution for literature search, writing, and correcting this minireview. All authors contributed to the article and approved the submitted version.

## Funding

The present work was supported by CONACYT scholarship #590338. 

## Conflict of Interest

The authors declare that the research was conducted in the absence of any commercial or financial relationships that could be construed as a potential conflict of interest.
